# Effects of Subacute Exposure of Dibutyl Phthalate on the Homeostatic Model Assessment, Thyroid Function, and Redox Status in Rats

**DOI:** 10.1155/2021/5521516

**Published:** 2021-08-04

**Authors:** Khalid Abdul Majeed, Muhammad Shahbaz Yousaf, Muhammad Sajid Tahir, Aamir Riaz Khan, Suliman Khan, Abdullah Arif Saeed, Habib Rehman

**Affiliations:** ^1^Department of Physiology, University of Veterinary and Animal Sciences, Lahore 54600, Pakistan; ^2^School of Chemistry and Molecular Biosciences, University of Queensland, Brisbane, Queensland, Australia 4072; ^3^Veterinary Research Institute, Lahore, Pakistan 54000; ^4^Department of Veterinary Physiology, Lasbela University of Agriculture, Water and Marine Sciences, Uthal, Balochistan 90150, Pakistan

## Abstract

Dibutyl phthalate is an endocrine disruptor used in a wide range of industrial and agriculture applications. The present study focuses on elucidating the effect of subacute exposure (4-weeks) of DBP on insulin and its sensitivity indexes, oxidative status, thyroid function, energy metabolites, serum biochemistry, and anthropometry in rats. A total of 64 rats were divided into 4 treatment groups as mg DBP/Kg body weight per day: (a) 0 mg/Kg (control), (b) 10 mg/Kg (DBP-10), (c) 50 mg/Kg (DBP-50), and (d) 100 mg/Kg (DBP-100). The rats in each treatment (*n* = 16) were further divided into male (*n* = 8) and female (*n* = 8) rats for studying treatment and gender interactions. Intraperitoneal glucose tolerance test (IPGTT) was performed on the 21^st^ day. Anthropometry, nutritional determinants, fasting plasma glucose, fasting plasma insulin, homeostatic model assessment (HOMA), thyroid hormones, energy metabolites, and oxidative status were studied during the experimental period. Two-way ANOVA was used to analyze the data (*p* < 0.05). Tukey's posthoc test was used for pair-wise comparisons. DBP increased body weight gain and feed efficiency in an inverted nonmonotonic *U*-shaped fashion. Hyperglycemia and increased blood glucose area under the curve were observed in DBP-100 at 120 minutes in IPGTT. The HOMA also showed a linear monotonic contrast. Thyroxin decreased significantly in the DBP-100 rats, whereas malondialdehyde, nonesterified fatty acids, and beta hydroxyl butyrate were increased with the DBP treatments. In conclusion, DBP could be attributed to the development of hyperglycemia and insulin resistance in rats. Further investigations into the lipid peroxidation pathways can improve our understanding of the mechanisms involved in metabolic disruption.

## 1. Introduction

Dibutyl phthalate (DBP) is a potent endocrine disruptor (ED) that can interfere with the homeostatic mechanisms of natural hormones. It is widely used in various everyday commercial products like cosmetics, varnishes, adhesives, plastic food wraps, inks, lacquers, and pharmaceuticals. Because of this extensive use, “One Health” is constantly exposed to the ill effects of DBP. The exposure is predominately through ingestion, inhalation, and absorption through the skin. DBP has contaminated food products like eggs [1], milk [2], meat [3], and drinking water ([4] [5]).

DBP belongs to phthalates that have the potential to interact with nuclear receptors PPARs (peroxisome proliferator-activated receptors) [6], the natural ligands of which are fatty acids and eicosanoids [7]. There is evidence that PPARs may be involved in chronic diseases like diabetes and obesity as reviewed [8]. Several epidemiological studies have also associated phthalates with diabetes. Urinary levels of phthalate metabolites are associated with prevalent diabetes in women [9], risk of type 2 diabetes in nurses [10], and increased waist circumference and insulin resistance in adult males [11] in the United States. Similarly, circulating levels of phthalates and their metabolites have been associated with diabetes prevalence in elderly individuals in Sweden [12] and glucose metabolism in peripubescent children in Mexico [13].

In experimental studies, DBP administered through oral route [14], inhalation [15], and intraperitoneal injection [16] has shown to increase the cytochrome P450 system that is associated with lipid and glucose homeostasis in rats. DBP has also been shown to disrupt evolutionarily conserved insulin and glucagon-like signaling pathways in Drosophila males [17]. Phthalates, other than DBP, have been shown to adversely affect the expression of insulin receptors and glucose transporter 4 (GLUT4) gene expression in myotubes [18]. There is a dire need to explore whether DBP can alter glucose metabolism and potentiate the risk of diabetes and insulin resistance. Insulin is a vital hormone involved in glucose uptake and inhibition of lipolysis in the adipose tissue through insulin receptors (IR) and its substrates (IRSs) [19]. The present study is designed to elucidate the effects of subacute exposure of DBP at above and below the no observed adverse effect level (NOAEL, DBP 50 mg/kg) on insulin, homeostatic model assessment (HOMA), thyroid function, and redox status in rats.

## 2. Materials and Methods

### 2.1. Experimental Animals

Sixty-four albino (32/gender) rats were used in the present study. The rats of similar anthropometry were housed in individual cages (Natsume Seisakusho Co. Ltd., Japan) at an experimental animal shed, Department of Physiology, University of Veterinary and Animal Sciences (UVAS), with free access to feed and water. The rats were maintained under 12-h light and 12-h dark cycle with an ambient temperature of 22 ± 3°C and relative humidity of 60-70%. Ethical commission for use of laboratory animals, UVAS, approved the experimental protocol (Directive No. DR/539).

### 2.2. Experimental Groups

The rats were offered standard rat chow (dietary component percentage by weight; dietary casein 20.5; glucose 20; cornstarch 39.7; cellulose 6; sunflower oil 5.8; vitamins 1; minerals 7). The rats were divided into four groups and administered DBP (Scharalu, Spain) in feed per kg body weight as (a) 0 mg/kg DBP per day (control), (b) 10 mg/kg DBP per day (DBP-10), (c) 50 mg/kg DBP per day (DBP-50), and (d) 100 mg/kg DBP per day (DBP-100) for a period of 4 weeks. Each group was further subdivided on the basis of gender (male/female). For administration via feed, DBP was solubilized in sunflower oil and mixed in feed.

### 2.3. Anthropometry and Nutritional Parameters

Body weight (g), body mass gain (g), feed consumption (g/day), energy intake (KJ/day), and feed efficiency (%) were measured for assessing nutritional parameters. Abdominal circumference (AC), thoracic circumference (TC), AC/TC ratio, body length, and body mass index (BMI) were measured to assess anthropometry. All these parameters were done as described previously [20, 21]. Relative organ weights (%) were calculated as described by [22].

### 2.4. Glucose Tolerance, Fasting Glucose, Fasting Insulin, and Insulin Sensitivity Indexes

Intraperitoneal glucose tolerance test was performed on the 21^st^ day after fasting rats overnight (5 pm to 8 am). After collecting a fasting blood glucose sample (0 minutes), a 10% glucose solution (1 g/Kg body weight) was injected intraperitoneally. This was followed by glucose measurements at 30, 60, and 120 minutes after injection. The glucose measurements were taken from tail blood using a blood glucose analyzer (Nipro Diagnostics, TRUEresult® Test strips, Lauderdale, US, Lot PT2857ITR, and PT2755ITR). The area under the blood glucose time-concentration curve was calculated as described previously [23]. At the end of the 4^th^ week, Fasting plasma glucose (Innoline, Merck, France) and insulin (Glory Bioscience, Del Rio, US, Lot 201506) were measured from serum collected at the end of the study (4^th^ week) on Epoch™ microplate reader (Biotek Instruments Inc. Winooski, US). Insulin sensitivity indexes were calculated as described previously [24].

### 2.5. Oxidative Stress, Thyroid Function, and Energy Metabolites

Oxidative damage was assessed by measuring serum catalase (BioVision, Inc. Milpitas, US, Lot 9E060773) and malondialdehyde (MDA) (BioVision, Inc. Milpitas, US, Lot 9E200739). Total triiodothyronine (T3) and thyroxin (T4) were estimated using BioCheck (Foster City, US, Lot RN-56911, and RN-56760) kits. For studying energy markers, nonesterified fatty acids (NEFA) (Randox, Crumlin, UK, Lot 350318) and beta-hydroxybutyrate (*β*-HB) (Pointe Scientific, Inc. Canton, US, Lot 633704-018) were estimated in serum.

### 2.6. Serum Chemistry

Serum chemistry included cholesterol, high-density lipoproteins (HDL), triglycerides, total proteins, albumin (A), globulin (G), and A/G ratio. Enzymes profile included lactate dehydrogenase (LDH), alkaline phosphatase (ALP), aspartate transaminase (AST), alanine transaminase (ALT), gamma-glutamyl transferase (GGT), and creatine kinase (CK). Renal function tests included blood urea nitrogen (BUN), serum creatinine, and uric acid. Commercial kits from Human Diagnostics (Wiesbaden, Germany) were used for all these parameters, except GGT (Chronolab, Barcelona, Spain) and CK (Innoline, Merck, France), and estimated on Epoch™ microplate spectrophotometer (Biotek Instruments Inc. Winooski, US).

### 2.7. Statistical Analysis

Data was checked for normality using the Kolmogorov Smirnov test. Data were analyzed by two-way ANOVA using SPSS software (SPSS Inc., Chicago, US, Version 16.0). The factors considered were treatment and gender. Homogeneity of variances was checked using Levene's test. *p* value less than 0.05 was considered statistically significant, and a *p* value less than 0.1 was considered a trend. Tukey's posthoc test was used for comparison between the treated groups. Polynomial contrasts were used to assess monotonic and nonmonotonic responses.

## 3. Results

The effects of subacute exposure of dibutyl phthalate on nutritional determinants and anthropometry are shown in Table 1. Body weight gain was affected in a *U*-shaped nonmonotonic response with an increase in DBP-10 compared with the control group (*p* = 0.02). Similarly, feed efficiency was also affected by an increase in DBP-10 compared with the control animals (*p* = <0.01). *U*-shaped nonmonotonic trends were observed for thoracic circumference (*p* = 0.06), abdominal circumference (*p* = 0.08), and body length (*p* = 0.08) with an increase in DBP-10 compared with the control rats. Relative weights of the liver and kidney increased with significance in the DBP-10 and DBP-100, respectively, compared with the control group (Table 2).

**Figure 1:**
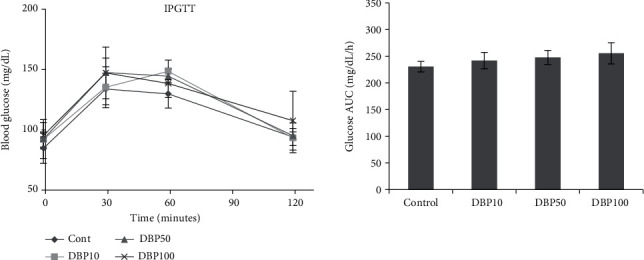
Blood glucose in intraperitoneal glucose tolerance test (IPGTT) and blood glucose area under the curve (AUC) in IPGTT. Two-way ANOVA was used for assessing the effects of treatment and gender.

A significant effect of DBP treatment (*p* = 0.017) and treatment into gender interaction (*p* = <0.01) was observed on blood glucose at 120 minutes in IPGTT. Similarly, a trend was observed for DBP treatment (*p* = 0.076) and treatment into gender interaction (*p* = 0.026) for blood glucose AUC (Figure 1). Considering DBP into gender interaction for blood glucose at 120 minutes in IPGTT, DBP-100 male was statistically different from males of the control, DBP-10, and DBP-50 (*p* = <0.01). Similarly, the control males were also statistically different from DBP-100 for blood glucose AUC (*p* = 0.05). No effects between the control and DBP-treated female rats were observed for IPGTT and AUC. An overall trend was observed for fasting glucose (*p* = 0.09). A linear monotonic response was observed for fasting glucose with a *p* value of 0.02. Similarly, the homeostatic model assessment of insulin resistance (HOMA-IR) was linearly significant with a *p* value of 0.05. No other effects of subacute exposure of dibutyl phthalate were observed on fasting insulin, quantitative insulin sensitivity check index (QUICIKI), and fasting glucose-insulin ratio (FGIR) (Table 3). No effects of the DBP treatment were observed on the IR (*p* = 0.9) and IRS-1 (*p* = 0.7) mRNA expression (Data not shown).

A trend (*p* = 0.1) of the decrease in total T3 was observed by the DBP treatments. However, a significant decrease was observed for total T4 compared with the control rats (*p* = 0.01). The serum catalase was not affected by any treatment. The serum MDA was increased significantly in the DBP-100 compared with the control rats (*p* = 0.01). The serum NEFA concentrations were increased in the DBP-10 rats compared with the control rats (*p* = 0.04). The serum *β*-HB was increased in all the DBP treated groups compared with the control group (*p* < 0.01) (Table 4). No effect of DBP treatment was observed on the lipid profile and serum proteins. However, a decreasing trend for linear contrast was observed for total proteins (*p* = 0.09) and globulins (*p* = 0.08). No effect was observed on BUN, serum creatinine, uric acid, ALT, AST, ALP, LDH, and GGT. A trend for serum CK was observed only (*p* = 0.06) (Table 5).

## 4. Discussion

Phthalates are extensively used in various commercial products and are therefore ubiquitous in our environment. DBP is an important phthalate, with a wide range of applications in various daily used products. Marsman [25] in detail has covered the effects of DBP in the national toxicology program technical reports. However, the doses used in that particular report were high. The traditional concept of dose-dependent hazardous effects has been questioned as several EDs produce toxic effects at low doses that are not observed at higher doses [26]. A similar low dose effect for DBP has been reported by Lee and Veeramachaneni [27], where DBP dose as low as 0.1 mg/Kg disrupted spermatogenesis in a frog. The present experiment determines the effects of subacute exposure of low concentrations of DBP on insulin and its sensitivity indexes, oxidative status, thyroid function, energy metabolites, serum chemistry, and anthropometry in rats.

No effect of DBP was observed on body weight. However, body weight gain increased significantly in lower doses (DBP-10), and a finding also observed in our subchronic exposure trial [28]. An increase in weight gain could be due to fat deposition because DBP can lead to PPAR pathway activation [29] which can cause adiposity. An inverted nonmonotonic response was observed for feed efficiency that relates directly to body weight gain. However, Marsman [25] and Ema et al. [30] have reported decreased body weight gain. This could be due to high exposure DBP doses of 250 mg/Kg and above. In females in the US, the metabolite of DBP, monobutyl phthalate (MBP), has been associated with faster prospective weight gains in a dose-response fashion [31]. No effects on anthropometry were observed, contrary to our subchronic exposure trail of DBP where the AC/TC ratio and the body mass index (BMI) were increased [28]. This could be clearly due to the length of exposure to DBP. A positive association has been reported between MBP, BMI, and circumference of the waist in Chinese and US studies in children [32, 33].

Blood glucose was higher at 120 minutes after IPGTT in DBP-100 males. Similarly, blood glucose AUC was also higher for DBP-100 males only compared with the control. Fasting plasma glucose showed a trend of increase in a linear monotonic fashion. The HOMA-IR also showed a linear monotonic significance. DBP is shown to cause hyperglycemia in *Drosophila* male flies by disrupting conserved insulin and glucagon-like signaling [17]. The HOMA-IR is an appropriate measure of insulin resistance in the development of type II diabetes [34]. DBP is also reported to have a strong influence on the synergism of action of a mixture of bisphenols A, S, and F, diethyl phthalate (DEP), and bisphenol A diglycidyl ether [35]. Therefore, the presence of DBP in the environment alongside other pollutants can augment the linear trend observed for fasting glucose and HOMA-IR (Table 3). In this context, combined effects of DBP and DEP have been shown to induce pancreatic beta-cell apoptosis in rat insulinoma cells [36].

The liver and kidney weights relative to body weight were increased in the DBP-10 and DBP-100 animals, respectively, compared with the control animals. However, no effects on liver enzymes and kidney function tests were observed in the present study of subacute exposure. The subchronic exposure of DBP had a negative impact on the liver and kidney functioning [28]. The present enlargement in the liver and kidney due to subacute exposure of DBP without changes in liver and kidney function tests is unclear. Histopathological examination of the liver and kidney can provide valuable information in this context. A trend for a decrease in total proteins (*p* = 0.09) and globulins (*p* = 0.08) was observed in a linear monotonic polynomial contrast. The decreased total proteins and globulins could be related to increased relative liver weight in the present study. A decreasing trend was observed in serum CK for the DBP-50 and DBP-100 exposed rats in a linear monotonic fashion. DBP could inhibit the acetylcholinesterase enzyme [37] and is known to increase tone in muscles [38]. The continuous exposure for 4 weeks could have led to a reduction in muscle mass that could have lowered serum CK, as is observed in alcoholics [39]. Also, subnormal levels of CK are observed in connective tissue disorders, even in the presence of histologically and clinically active myositis [40].

In this study, DBP decreased total T4 and showed a trend of decrease in total T3. The findings are consistent with a high dose DBP exposure [41] and several epidemiological reports [42, 43]. The decrease can be attributed to a disruption in thyroid signaling, as DBP and its metabolite have been shown to possess thyroid hormone receptor antagonistic activities in *Xenopus laevis* [44]. Low doses of DBP are shown to aggravate chronic lymphocytic thyroiditis, a common autoimmune disorder through increased oxidative stress [45]. Oxidative stress through reactive oxygen species is important in autoimmune conditions because their accumulation may be toxic to the DNA. The serum catalase was not affected but serum MDA was significantly increased in the DBP-100 rats compared with the control rats. Testicular catalase has been shown to decrease in rats after exposure to DBP [46]. On the contrary, DBP increased the catalase and MDA levels in Wistar rats [47] and in *Karenia brevis* algae [48]. Recently, a mixture of DBP along with bisphenol A and DEP has been shown to change the redox status in the rat pancreatic tissue [49]. An insight into the intermediate products of lipid peroxidation and enzymes that generate these products could provide fruitful information for the present study. A similar mechanism of lipid peroxidation due to deleterious effects of reactive oxygen species on spermatozoa has been reported by Baralić et al. [50] where MDA levels were increased after exposure to DBP, DEP, and bisphenol A. Increased NEFA concentrations were observed in DBP-10, whereas *β*-HB was increased in all the DBP-treated groups. Sustained reduction in systemic NEFA has been shown to improve insulin sensitivity without restoration of the oxidative capacity of mitochondria [51]. Diabetes is an important cause of elevated blood ketones that includes *β*-HB, acetoacetate, and acetone leading to ketoacidosis [52]. Acetoacetate, but not *β*-HB, has been shown to cause an increase in lipid peroxidation [53].

In conclusion, the DBP could be attributed to the development of hyperglycemia and insulin resistance in rats. Further investigations into the lipid peroxidation pathways can improve our understanding of the mechanisms involved in metabolic disruption.

## Figures and Tables

**Table 1:** Effects of subacute exposure of dibutyl phthalate on nutritional determinants and anthropometric parameters of obesity in rats.

	Control	DBP 10 mg/kg	DBP 50 mg/kg	DBP 100 mg/kg	*p* (treatment)	*p* (linear)	*p* (quadratic)	*p* (treatment×gender)
Body weight (g)	187.7 ± 9.5	205.5 ± 14.9	204.7 ± 15.6	192.0 ± 13.3	0.32	0.84	0.27	0.20
Body weight gain (g)	61.5 ± 7.0^a^	95.5 ± 9.8^b^	76.0 ± 11.0^ab^	64.0 ± 12.2^a^	0.02	0.79	0.03	0.45
Feed consumed (g/day)	18.2 ± 0.57	18.7 ± 0.56	18.8 ± 0.56	18.5 ± 0.7	0.90	0.75	0.60	0.80
Energy intake (KJ/day)	266.57 ± 8.4	272.12 ± 8.3	273.8 ± 8.3	270.5 ± 10.3	0.90	0.74	0.60	0.84
Feed efficiency (%)	6.4 ± 0.58^a^	8.5 ± 0.33^b^	8.1 ± 0.45^bc^	6.8 ± 0.50^ac^	<0.01	0.90	<0.01	0.60
Thoracic circumference (cm)	11.7 ± 0.23	12.5 ± 0.34	12.4 ± 0.32	12.0 ± 0.24	0.19	0.54	0.06	0.56
Abdominal circumference (cm)	12.7 ± 0.21	13.7 ± 0.43	13.5 ± 0.33	13.3 ± 0.18	0.14	0.32	0.08	0.30
AC/TC	1.08 ± 0.01	1.09 ± 0.01	1.09 ± 0.01	1.1 ± 0.02	0.46	0.12	0.87	0.21
Body length (cm)	18.8 ± 0.54	19.6 ± 0.59	19.6 ± 0.60	18.5 ± 0.16	0.11	0.69	0.08	0.21
BMI (g/cm^2^)	0.53 ± 0.02	0.53 ± 0.02	0.53 ± 0.02	0.55 ± 0.04	0.58	0.39	0.52	0.69

Data are presented as mean ± standard error. Superscripts ^a–c^ within the row indicate significant differences (*p* < 0.05). Two-way ANOVA was used for assessing the effects of treatment and gender. Polynomial contrasts (linear and quadratic) were used to assess monotonic and nonmonotonic responses.

**Table 2:** Effects of subacute exposure of dibutyl phthalate on relative organ weights (%) in rats.

	Control	DBP 10 mg/kg	DBP 50 mg/kg	DBP 100 mg/kg	*p* (treatment)	*p* (linear)	*p* (quadratic)	*p* (treatment×gender)
Liver	3.01 ± 0.16^a^	3.48 ± 0.12^b^	3.35 ± 0.08^ab^	3.43 ± 0.1^ab^	0.03	0.04	0.11	0.41
Kidney	0.73 ± 0.02^a^	0.78 ± 0.02^ab^	0.80 ± 0.02^ab^	0.86 ± 0.04^b^	0.01	<0.01	0.81	0.20
Heart	0.33 ± 0.01	0.34 ± 0.01	0.29 ± 0.01	0.31 ± 0.01	0.10	0.17	0.63	0.51
Lungs	0.60 ± 0.04	0.64 ± 0.04	0.61 ± 0.03	0.63 ± 0.04	0.88	0.74	0.78	0.59
Spleen	0.21 ± 0.01	0.22 ± 0.01	0.24 ± 0.01	0.23 ± 0.01	0.19	0.15	0.47	0.45

Data are presented as mean ± standard error. Superscripts ^a-b^ within the row indicate significant differences (*p* < 0.05). Two-way ANOVA was used for assessing the effects of treatment and gender. Polynomial contrasts (linear and quadratic) were used to assess monotonic and nonmonotonic responses.

**Table 3:** Effects of subacute exposure of dibutyl phthalate on fasting glucose, insulin, and insulin sensitivity indexes in rats.

	Control	DBP 10 mg/kg	DBP 50 mg/kg	DBP 100 mg/kg	*p* (treatment)	*p* (linear)	*p* (quadratic)	*p* (treatment×gender)
Fasting glucose (mg/dL)	89.2 ± 2.5	93.6 ± 2.1	98.8 ± 3.1	97.2 ± 3.0	0.09	0.02	0.28	0.25
Fasting insulin (*μ*U/mL)	10.1 ± 1.0	8.7 ± 0.6	10.2 ± 0.8	10.7 ± 1.1	0.28	0.38	0.25	0.20
HOMA-IR	0.35 ± 0.03	0.35 ± 0.02	0.43 ± 0.04	0.45 ± 0.06	0.13	0.05	0.87	0.21
QUICKI	0.34 ± 0.01	0.34 ± 0.01	0.33 ± 0.01	0.33 ± 0.01	0.30	0.12	0.91	0.22
FGIR (mg/10^−4^ U)	10.0 ± 0.83	10.2 ± 0.68	10.0 ± 0.73	8.6 ± 1.2	0.52	0.26	0.41	0.20

Data are presented as mean ± standard error. Two-way ANOVA was used for assessing the effects of treatment and gender. Polynomial contrasts (linear and quadratic) were used to assess monotonic and nonmonotonic responses.

**Table 4:** Effect of subacute exposure of dibutyl phthalate on thyroid hormones, oxidative stress, and energy metabolites in rats.

	Control	DBP 10 mg/kg	DBP 50 mg/kg	DBP 100 mg/kg	*p* (treatment)	*p* (linear)	*p* (quadratic)	*p* (treatment × gender)
Total T3 (ng/dL)	97.15 ± 0.2	97.12 ± 0.6	95.14 ± 0.9	96.83 ± 0.8	0.10	0.71	0.19	0.38
Total T4 (*μ*g/dL)	8.27 ± 0.4^a^	7.11 ± 0.3^ab^	6.94 ± 0.5^ab^	5.79 ± 0.8^b^	0.01	<0.01	0.90	0.21
Catalase activity (nmol/min/mL)	166.5 ± 19.6	140.8 ± 17.6	144.9 ± 13.0	122.5 ± 12.5	0.79	0.39	0.91	0.23
Malondialdehyde (nmol/mL)	1.38 ± 0.03^a^	1.87 ± 0.1^ab^	1.97 ± 0.2^ab^	2.23 ± 0.2^b^	0.01	0.01	0.56	0.66
NEFA (mmol/L)	0.81 ± 0.04^a^	1.28 ± 0.1^b^	1.09 ± 0.04^ab^	1.07 ± 0.08^ab^	0.04	0.13	0.02	0.20
*β*-HB (mmol/L)	0.87 ± 0.03^a^	1.09 ± 0.03^b^	1.05 ± 0.03^b^	1.04 ± 0.04^b^	< 0.01	0.03	0.10	0.24

Data are presented as mean ± standard error. Superscripts ^a-b^ within the row indicate significant differences (*p* < 0.05). Two-way ANOVA was used for assessing the effects of treatment and gender. Polynomial contrasts (linear and quadratic) were used to assess monotonic and nonmonotonic responses.

**Table 5:** Effects of subacute exposure of dibutyl phthalate on the serum biochemistry in rats.

	Control	DBP 10 mg/kg	DBP 50 mg/kg	DBP 100 mg/kg	*p* (treatment)	*p* (linear)	*p* (quadratic)	*p* (treatment × gender)
Cholesterol (mg/dL)	86.91 ± 5.3	76.35 ± 4.2	81.47 ± 4.5	84.70 ± 4.8	0.18	0.88	0.14	0.65
Triglycerides (mg/dL)	114.54 ± 4.4	113.08 ± 4.7	118.79 ± 1.7	114.51 ± 4.1	0.60	0.75	0.71	0.20
HDL (mg/dL)	44.46 ± 1.2	44.57 ± 2.2	35.82 ± 5.1	38.25 ± 5.5	0.40	0.15	0.74	0.43
Total protein (g/dL)	6.92 ± 0.5	6.19 ± 0.4	6.18 ± 0.3	5.95 ± 0.2	0.28	0.09	0.51	0.17
Albumin (g/dL)	3.29 ± 0.2	2.91 ± 0.1	3.05 ± 0.1	3.27 ± 0.1	0.16	0.89	0.13	0.20
Globulin (g/dL)	3.01 ± 0.4	2.72 ± 0.3	2.62 ± 0.3	2.24 ± 0.2	0.26	0.08	0.88	0.21
A/G ratio	1.75 ± 0.7	1.16 ± 0.1	1.25 ± 0.1	1.54 ± 0.1	0.58	0.73	0.22	0.25
BUN (mg/dL)	24.75 ± 1.6	24.95 ± 1.2	25.10 ± 2.2	21.66 ± 1.4	0.29	0.22	0.27	0.28
Creatinine (mg/dL)	0.78 ± 0.1	0.85 ± 0.2	0.98 ± 0.2	0.85 ± 0.1	0.77	0.61	0.50	0.33
Uric acid (mg/dL)	5.86 ± 0.2	5.63 ± 0.3	5.69 ± 0.2	5.03 ± 0.1	0.13	0.12	0.46	0.17
ALT (U/L)	39.16 ± 3.8	32.40 ± 3.0	37.58 ± 3.7	42.11 ± 6.4	0.49	0.43	0.20	0.83
AST (U/L)	86.95 ± 3.5	78.55 ± 5.8	86.65 ± 5.8	95.46 ± 7.6	0.48	0.23	0.14	0.81
ALP (U/L)	96.42 ± 12.6	128.16 ± 14.5	139.26 ± 18.4	133.04 ± 16.1	0.53	0.22	0.35	0.71
LDH (U/L)	442.50 ± 51.1	510.83 ± 29.8	464.25 ± 44.0	513.33 ± 37.6	0.78	0.51	0.88	0.67
GGT (U/L)	2.04 ± 0.4	2.40 ± 0.4	1.86 ± 0.2	1.93 ± 0.2	0.74	0.61	0.69	0.43
CK (U/L)	420.75 ± 63.7	464.8 ± 48.9	302.7 ± 32.7	283.8 ± 23.63	0.06	0.03	0.58	0.19

Data are presented as mean ± standard error. Two-way ANOVA was used for assessing the effects of treatment and gender. Polynomial contrasts (linear and quadratic) were used to assess monotonic and nonmonotonic responses.

## Data Availability

Data is available through institutional review board, Department of Physiology, UVAS.
